# Supplementary material for: Assessing B-Z DNA Transitions in Solutions via Infrared Spectroscopy
Source: Biomolecules. 2023 Jun 8;13(6):964. doi: 10.3390/biom13060964 (PMC10295956; doi:10.3390/biom13060964)
Supplement: Supplementary file 1 [file biomolecules-13-00964-s001.zip › biomolecules-2376213-supplementary.pdf]

Supplementary Materials

Assessing B-Z DNA Transitions in Solutions via Infrared Spectroscopy

Mengmeng Duan [1], Yalin Li [2], Fengqiu Zhang [1,*] and Qing Huang [3,4,*]

[1] Henan Key Laboratory of Ion-Beam Bioengineering, School of Physics and Microelectronics, Zhengzhou University, Zhengzhou 450052, China; 202012132012347@gs.zzu.edu.cn

[2] School of Food and Biological Engineering, Henan University of Animal Husbandry and Economy, Zhengzhou 450047, China; 201087@hnuahe.edu.cn

[3] CAS Key Laboratory of High Magnetic Field and Ion Beam Physical Biology, Institute of Intelligent Machines, Hefei Institutes of Physical Sciences, Chinese Academy of Sciences, Hefei 230031, China

[4] Science Island Branch of Graduate School, University of Science and Technology, Hefei 230026, China

* Correspondence: zhangfengqiu@zzu.edu.cn (F.Z.); huangq@ipp.ac.cn (Q.H.)

1. Spectral analysis of Z-DNA induced by NaCl

1.1 Infrared spectral fitting of Z-DNA induced by NaCl

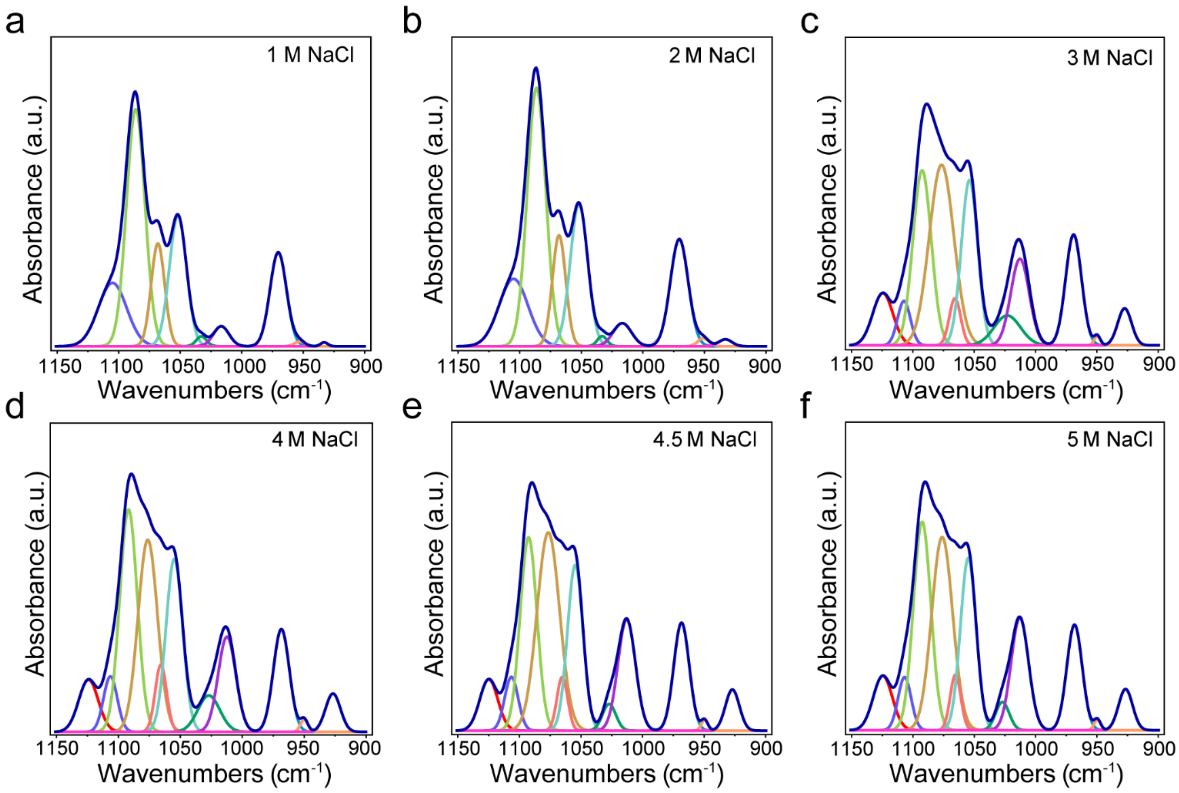

Figure S1. Fitting results of representative infrared spectra of dsDNA induced by different concentrations of NaCl in the range of 1150 cm^{-1}–900 cm^{-1}.

The second derivative processing of the infrared spectrum was carried out to obtain the peak position for peak division, and Origin software was used for Gaussian fitting of the spectrum.

Table S1. Fitting parameters of infrared spectra of dsDNA treated by NaCl.

Peak	Fit parameters	NaCl					
		1 M	2 M	3 M	4 M	4.5 M	5 M
1	x_c (cm^{-1})	-	-	1124.45	1123.316	1123.259	1122.903
	w (cm^{-1})	-	-	16.467	17.525	18.572	18.851
	A/A_{970}	-	-	0.6	0.683	0.701	0.766
2	x_c (cm^{-1})	1105.049	1105	1107.211	1105.913	1105.478	1105.821
	w (cm^{-1})	23.051	23.497	10.464	10.402	9.434	9.151
	A/A_{970}	1.16	1.021	0.323	0.409	0.33	0.309
3	x_c (cm^{-1})	1086.131	1086.303	1092.437	1091.818	1092.603	1092.52
	w (cm^{-1})	15.018	15.047	14.963	14.193	13.618	14.231
	A/A_{970}	2.826	2.505	1.824	2.375	1.943	2.244
4	x_c (cm^{-1})	-	-	1076.549	1076.252	1076.527	1076.174
	w (cm^{-1})	-	-	20.093	15.75	18.407	17.121
	A/A_{970}	-	-	2.527	2.277	2.693	2.503
5	x_c (cm^{-1})	1068.116	1068.036	1065.616	1065.693	1065.217	1065.34
	w (cm^{-1})	10.985	10.84	9.241	9.208	8.667	8.641
	A/A_{970}	0.897	0.775	0.303	0.465	0.343	0.363
6	x_c (cm^{-1})	1051.827	1051.832	1053.77	1054.846	1054.675	1054.879
	w (cm^{-1})	13.962	14.151	13.317	13.467	12.613	12.836
	A/A_{970}	1.447	1.299	1.534	1.753	1.542	1.671
7	x_c (cm^{-1})	1032.77	1033.035	1023	1027	1027.153	1027.061
	w (cm^{-1})	8.408	7.842	21.491	17.734	10.826	11.039
	A/A_{970}	0.066	0.051	0.446	0.485	0.213	0.236
8	x_c (cm^{-1})	1016.647	1016.692	1012.639	1012.342	1012.88	1012.85
	w (cm^{-1})	12.657	14.848	15.03	14.27	14.223	14.289
	A/A_{970}	0.201	0.221	0.905	1.021	1.169	1.215
9	x_c (cm^{-1})	970.309	970.423	968.945	968.412	968.351	968.234
	w (cm^{-1})	13.414	14.484	12.961	12.934	12.555	12.526
	A/A_{970}	1	1	1	1	1	1
10	x_c (cm^{-1})	952.889	950.897	949.638	950.027	949.622	949.606
	w (cm^{-1})	8.445	8.867	5.007	5.964	5.304	5.329
	A/A_{970}	0.032	0.045	0.032	0.055	0.041	0.043
11	x_c (cm^{-1})	933	933	927.318	926.759	927.04	926.608
	w (cm^{-1})	5.734	10.68	11.255	12.055	11.322	11.486
	A/A_{970}	0.018	0.047	0.29	0.347	0.344	0.358

Note: "-" means there is no peak. x_c represents the wavenumber, w represents the bandwidth, A represents the integrated intensity, and A_{970} represents the internal parameter standard.

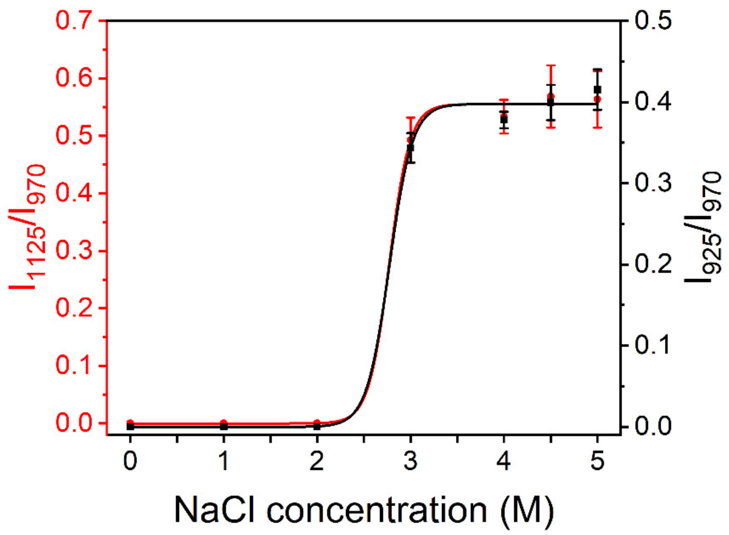

Figure S2. Changes of the ratio of Z-DNA characteristic peaks (925 cm^{-1} and 1125 cm^{-1}) intensity to 970 cm^{-1} peak intensity with NaCl treatment.

The fitting function: $y=A_2+(A_1-A_2)/(1+exp((x-x_0)/dx))$

I_{1125}/I_{970}:

Reduced Chi-Sqr 2.41574E-4

Adj. R-Square 0.99712

I_{925}/I_{970}:

Reduced Chi-Sqr 2.33505E-4

Adj. R-Square 0.99452

1.2 CD spectra of DNA

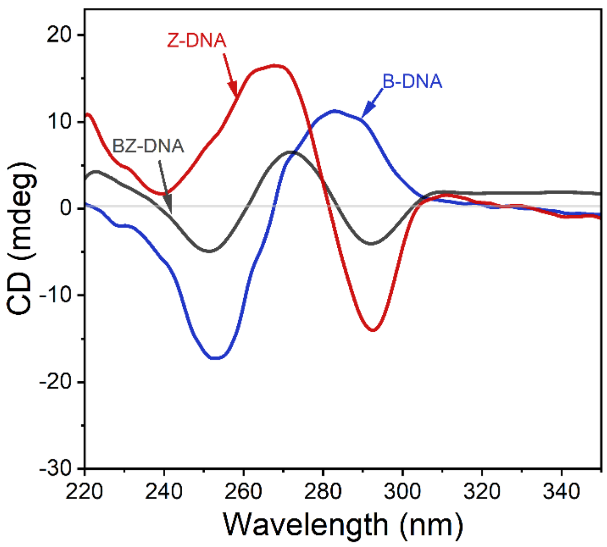

Figure S3. CD spectra for identification of $d(GC)_8$ chiral structures.

1.3 Analysis of Z-DNA induced by NaCl through ultraviolet spectroscopy

The instrument used in this work is NanoDrop 2000 ultramicro spectrophotometer (Thermo Scientific, USA). The detection temperature was at room temperature, and ultrapure water was used as a blank control. All nucleic acid samples were kept at the same concentration during detection, and each treatment was set up in triplicate.

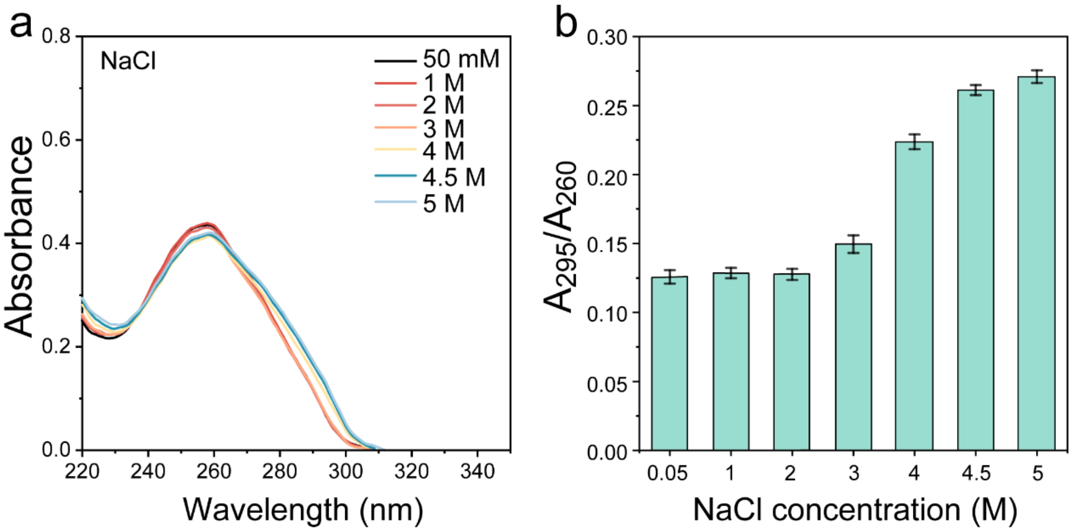

Figure S4. UV analysis of d(GC)$_8$ dsDNA treated with different concentrations of NaCl. a) UV absorption spectra of DNA treated with different concentrations of NaCl; b) Analysis of A_{295}/A_{260} of DNA UV absorption after different NaCl treatments.

2. Spectral analysis of Z-DNA induced by $MgCl_2$

2.1 Infrared spectral fitting of Z-DNA induced by $MgCl_2$

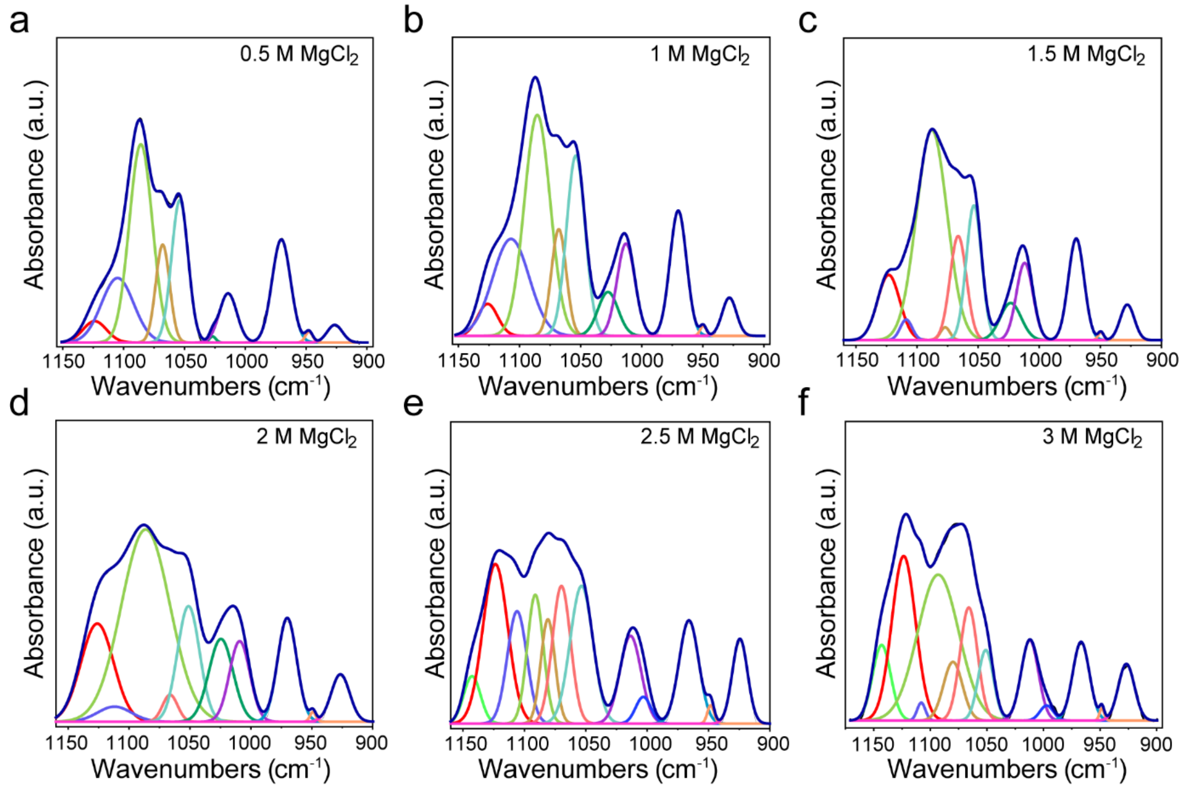

Figure S5. Fitting results of representative infrared spectra of dsDNA induced by different concentrations of $MgCl_2$ in the range of 1150 cm^{-1}–900 cm^{-1}.

The second derivative processing of the infrared spectrum was carried out to obtain the peak position for peak division, and Origin software was used for Gaussian fitting of the spectrum.

Table S2. Fitting parameters of infrared spectra of dsDNA treated by $MgCl_2$.

Peak	Fit parameters	$MgCl_2$					
		0.5 M	1 M	1.5 M	2 M	2.5 M	3 M
1	x_c (cm^{-1})	-	-	-	-	1142.614	1143
	w (cm^{-1})	-	-	-	-	13.75	16.027
	A/A_{970}	-	-	-	-	0.394	1.043
2	x_c (cm^{-1})	1123.766	1126.134	1123.226	1126.13	1123.653	1124.424
	w (cm^{-1})	19.424	16.437	18.283	26.775	20.321	19.839
	A/A_{970}	0.264	0.309	0.910	1.618	1.961	2.76
3	x_c (cm^{-1})	1104.985	1107.029	1108.885	1112	1105.96	1109.614
	w (cm^{-1})	26.459	29.754	9.82	27.679	15.853	12.196
	A/A_{970}	1.087	1.697	0.154	0.257	1.078	0.318
4	x_c (cm^{-1})	1085.858	1085.499	1087.929	1086.381	1091.117	1093
	w (cm^{-1})	19.044	21.274	25.115	40.011	13.095	36.437
	A/A_{970}	2.404	2.765	4.028	4.739	1.021	4.579
5	x_c (cm^{-1})	-	-	-	-	1080.84	1080
	w (cm^{-1})	-	-	-	-	11.511	16.843
	A/A_{970}	-	-	-	-	0.726	0.851
6	x_c (cm^{-1})	-	-	1077	-	-	-
	w (cm^{-1})	-	-	7.849	-	-	-
	A/A_{970}	-	-	0.077	-	-	-
7	x_c (cm^{-1})	1067.9	1067.9	1066.324	1066.374	1069.841	1066
	w (cm^{-1})	11.414	12.135	13.44	12.341	14.875	15.466
	A/A_{970}	0.711	0.761	1.068	0.205	1.239	1.505
8	x_c (cm^{-1})	1053.82	1053.92	1053.438	1051.199	1053.453	1051
	w (cm^{-1})	13.667	15.207	12.591	18.187	18.555	13.472
	A/A_{970}	1.238	1.613	1.295	1.295	1.546	0.818
9	x_c (cm^{-1})	1026.9	1027.608	1023.372	1024.565	1013.615	1012.503
	w (cm^{-1})	5.211	16.463	17.243	19.875	17.104	14.843
	A/A_{970}	0.02	0.425	0.49	1.014	0.908	1.008
10	x_c (cm^{-1})	1014.151	1012.907	1011.96	1009.194	1003.179	998.663
	w (cm^{-1})	14.418	14.721	13.536	16.722	12.398	13.075
	A/A_{970}	0.451	0.799	0.798	0.83	0.2	0.201
11	x_c (cm^{-1})	970.383	970.119	969.745	970.141	966.02	966.812
	w (cm^{-1})	15.234	13.569	12.888	15.636	16.005	14.749
	A/A_{970}	1	1	1	1	1	1
12	x_c (cm^{-1})	947.6465	949.619	949.524	949.043	948.287	948.415
	w (cm^{-1})	6.06902	5.107	4.547	4.846	5.4154	4.13
	A/A_{970}	0.042	0.03	0.028	0.029	0.06	0.043
13	x_c (cm^{-1})	926.462	928.282	927.961	926.458	924.507	926.764
	w (cm^{-1})	12.535	11.673	11.562	15.129	12.435	14.199
	A/A_{970}	0.14	0.262	0.314	0.442	0.638	0.693

Note: "-" means there is no peak. x_c represents the wavenumber, w represents the bandwidth, A represents the integrated intensity, and A_{970} represents the internal parameter standard.

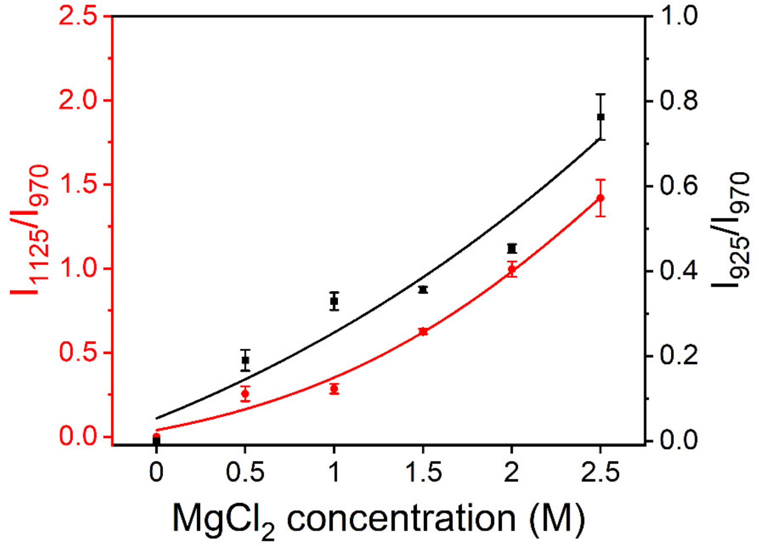

Figure S6. Changes of the ratio of the Z-DNA characteristic peaks (925 cm^{-1} and 1125 cm^{-1}) intensity to 970 cm^{-1} peak intensity after $MgCl_2$ treatment.

The fitting function: $y=A_2+(A_1-A_2)/(1+\exp((x-x_0)/dx))$

I_{1125}/I_{970}:

Reduced Chi-Sqr 0.00293

Adj. R-Square 0.98956

I_{925}/I_{970}:

Reduced Chi-Sqr 0.00413

Adj. R-Square 0.93731

2.2 Analysis of Z-DNA induced by $MgCl_2$ through ultraviolet spectroscopy

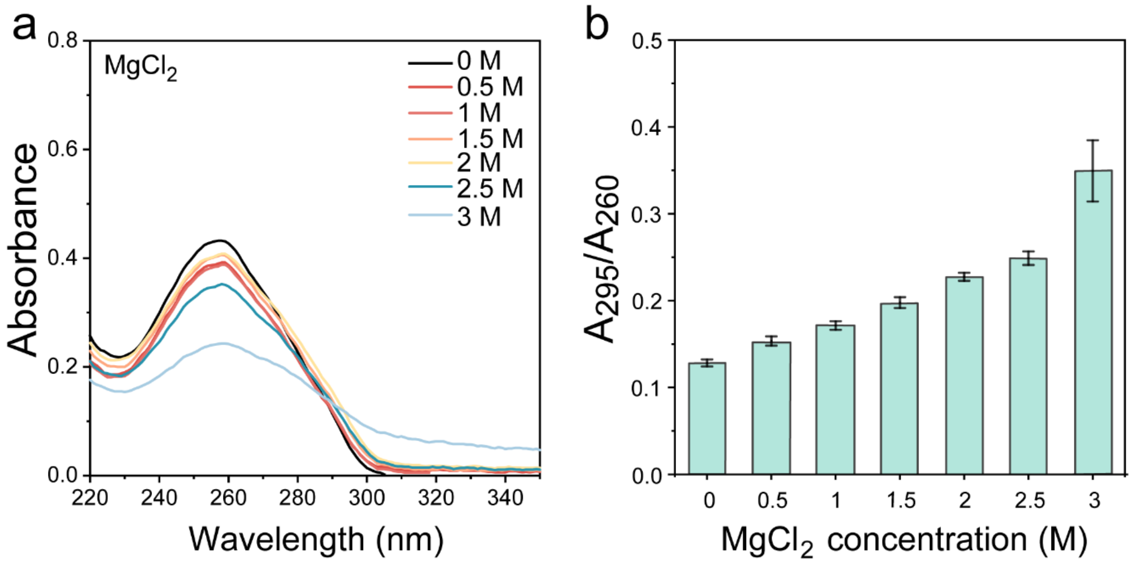

Figure S7. UV analysis of $d(GC)_8$ dsDNA treated with different concentrations of $MgCl_2$. a) The UV absorption spectra of DNA treated with different concentrations of $MgCl_2$; b) The analysis of A_{295}/A_{260} of DNA UV absorption after different concentrations of $MgCl_2$ treatments.

3. Spectral analysis of Z-DNA induced by ethanol

3.1 Infrared spectral fitting of Z-DNA induced by ethanol

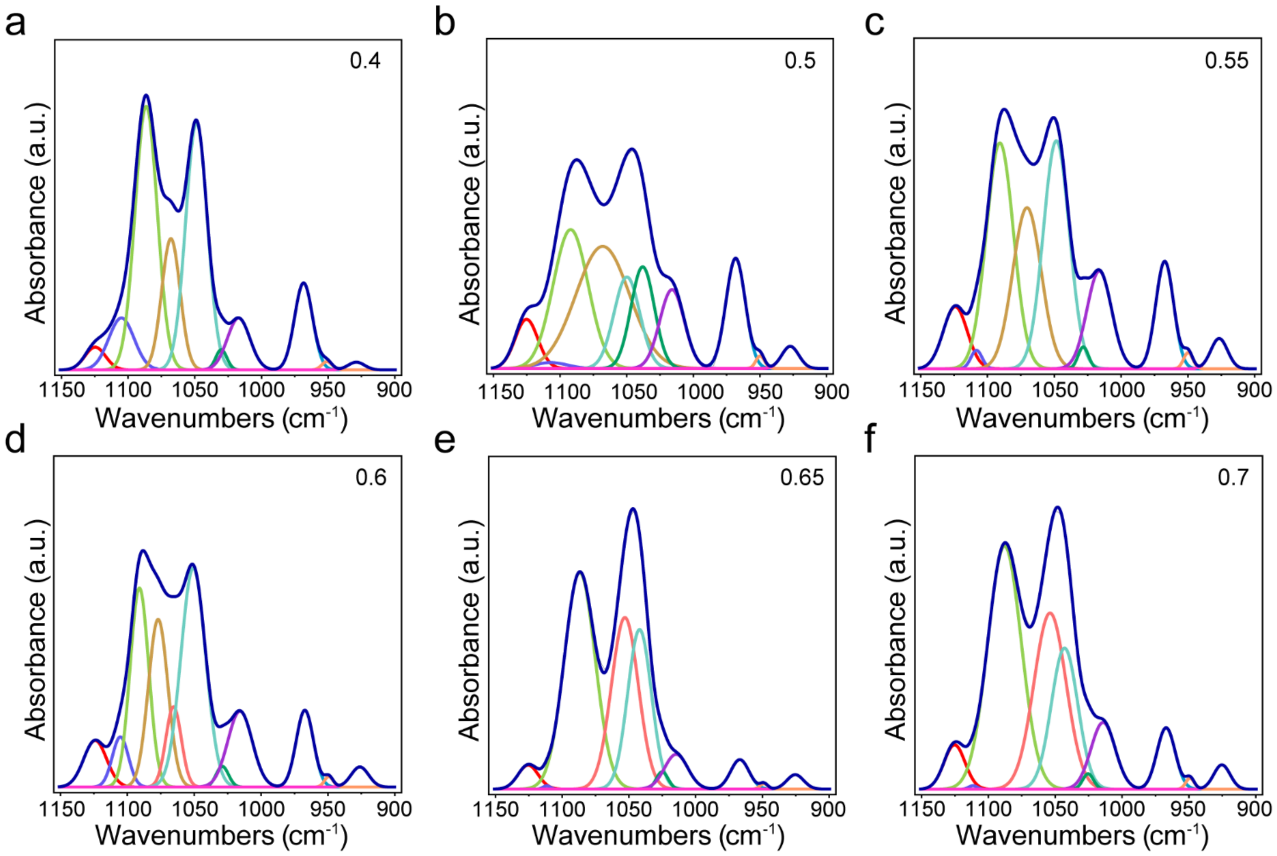

Figure S8. Fitting results of representative infrared spectra of dsDNA induced by ethanol of different volume ratios in the range of 1150 cm^{-1}–900 cm^{-1}.

The second derivative processing of the infrared spectrum was carried out to obtain the peak position for peak division, and Origin software was used for Gaussian fitting of the spectrum.

Table S3. Fitting parameters of infrared spectra of dsDNA treated by ethanol.

Peak	Fit parameters	Ethanol (v/v)					
		0.4	0.5	0.55	0.6	0.65	0.7
1	x_c (cm^{-1})	1124.66	1125.202	1124.092	1124.079	1125.497	1125.302
	w (cm^{-1})	15.449	17.054	17.969	17.121	15.064	15.749
	A/A_{970}	0.299	0.522	0.774	0.805	0.891	0.831
2	x_c (cm^{-1})	1105	1107	1108.202	1105.225	1111.61	1111.529
	w (cm^{-1})	18.999	24.146	8.361	12.11	5.309	6.327
	A/A_{970}	0.842	0.089	0.107	0.613	0.042	0.026
3	x_c (cm^{-1})	1086.43	1092.057	1090.704	1090.976	1086.835	1087.909
	w (cm^{-1})	16.686	26.183	20.333	13.916	23.117	25.075
	A/A_{970}	3.751	2.264	3.187	2.793	12.406	7.254
4	x_c (cm^{-1})	1067.9	1068	1070.411	1076.965	-	-
	w (cm^{-1})	13.779	40.237	21.317	14.643	-	-
	A/A_{970}	1.543	3.059	2.384	2.478	-	-
5	x_c (cm^{-1})	-	-	-	1065.447	1053	1053.863
	w (cm^{-1})	-	-	-	11.561	20.199	24.034
	A/A_{970}	-	-	-	0.937	8.579	5.037
6	x_c (cm^{-1})	1048.692	1049.844	1048.442	1050.697	1042	1043

	w (cm^{-1})	16.214	19.387	19.472	18.468	17.684	19.763
	A/A_{970}	3.418	1.104	3.08	4.079	6.985	3.316
7	x_c (cm^{-1})	1025.507	1038	1028.281	1029.015	1025.037	1025.388
	w (cm^{-1})	14.116	17.543	7.74	9.235	8.747	8.046
	A/A_{970}	0.479	1.11	0.117	0.194	0.374	0.147
8	x_c (cm^{-1})	1012.857	1015.997	1016.323	1015.766	1014.601	1014.182
	w (cm^{-1})	12.858	18.366	18.476	18.267	17.438	18.205
	A/A_{970}	0.415	0.902	1.254	1.399	1.500	1.445
9	x_c (cm^{-1})	968.406	968.225	967.351	967.261	966.883	967.056
	w (cm^{-1})	13.476	14.566	13.402	12.901	13.73	13.675
	A/A_{970}	1	1	1	1	1	1
10	x_c (cm^{-1})	950.93	949.809	949.781	949.372	948.854	949.188
	w (cm^{-1})	7.774	6.54	6.878	6.57	5.74	6.371
	A/A_{970}	0.055	0.051	0.077	0.069	0.078	0.083
11	x_c (cm^{-1})	928.878	927.26	926.609	926.156	925.368	925.324
	w (cm^{-1})	12.269	12.771	12.565	13.089	12.531	12.236
	A/A_{970}	0.081	0.176	0.264	0.266	0.445	0.355

Note: "-" means there is no peak. x_c represents the wavenumber, w represents the bandwidth, A represents the integrated intensity, and A_{970} represents the internal parameter standard.

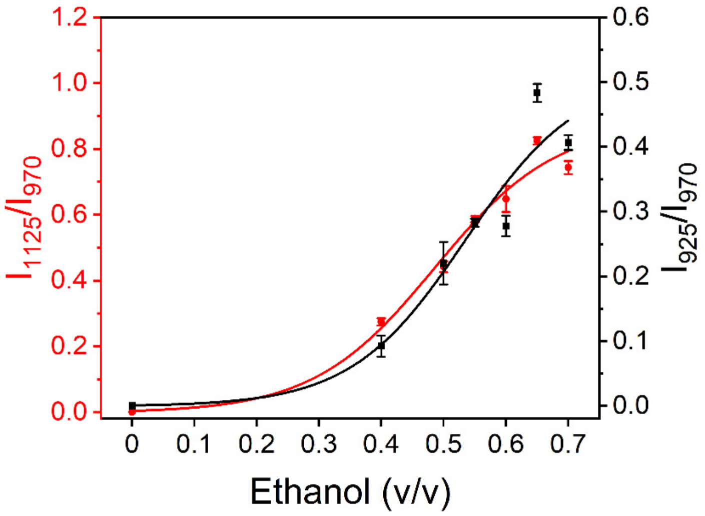

Figure S9. Changes of the ratio of the Z-DNA characteristic peaks (925 cm^{-1} and 1125 cm^{-1}) intensity to 970 cm^{-1} peak intensity after ethanol treatment.

The fitting function: $y=A_2+(A_1-A_2)/(1+exp((x-x_0)/dx))$

I_{1125}/I_{970}:

Reduced Chi-Sqr 0.00182

Adj. R-Square 0.97806

I_{925}/I_{970}:

Reduced Chi-Sqr 0.00213

Adj. R-Square 0.92453

3.2 Analysis of Z-DNA induced by ethanol through ultraviolet spectroscopy

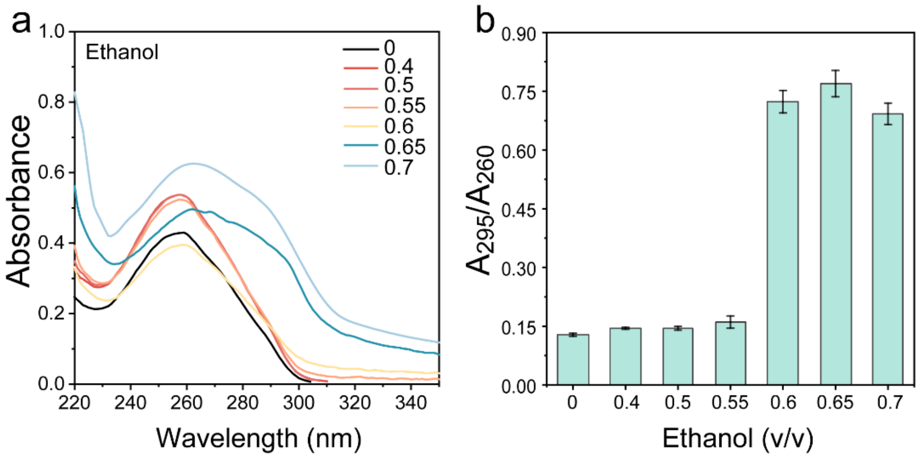

Figure S10. UV analysis of d(GC)$_8$ dsDNA after ethanol treatment at different volume ratios. a) The UV absorption spectra of DNA treated with different volume ratios of ethanol; b) The analysis of A_{295}/A_{260} of DNA UV absorption after different ethanol treatments.

4. Changes analysis of 1016 cm^{-1} peak in FTIR spectra

In addition to the Z-DNA characteristic peaks of 1125 cm^{-1}and 925 cm^{-1}, furanose 1016 cm^{-1} also changed with the concentration changes of inducers.

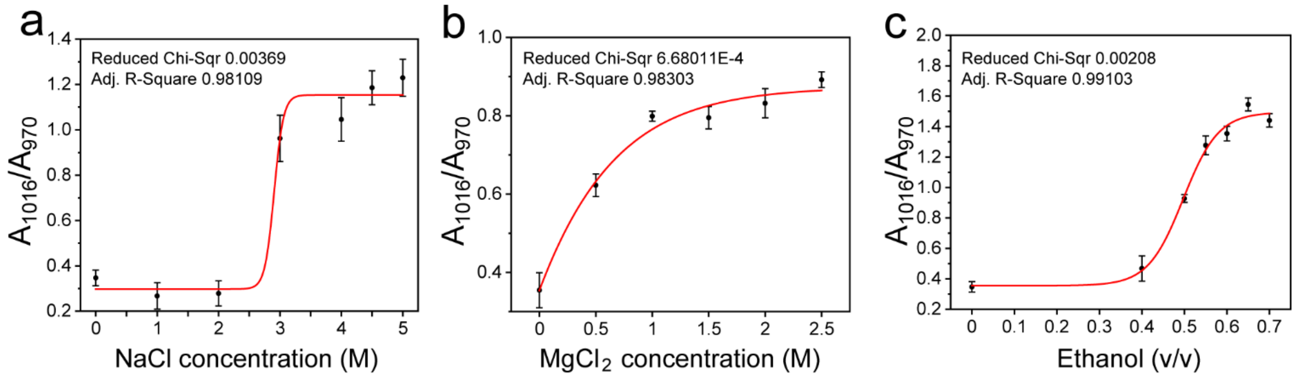

Figure S11. The ratio changes of 1016 cm^{-1} peak area to 970 cm^{-1} peak area with the concentration of inducers (data were supplied in the spectral fitting). a) The changes of A_{1016}/A_{970} of d(GC)$_8$ dsDNA induced by NaCl; b) The changes of A_{1016}/A_{970} of d(GC)$_8$ dsDNA induced by $MgCl_2$; c) The changes of A_{1016}/A_{970} of d(GC)$_8$ dsDNA induced by ethanol. The fitting function: $y=A_2+(A_1-A_2)/(1+\exp((x-x_0)/dx))$

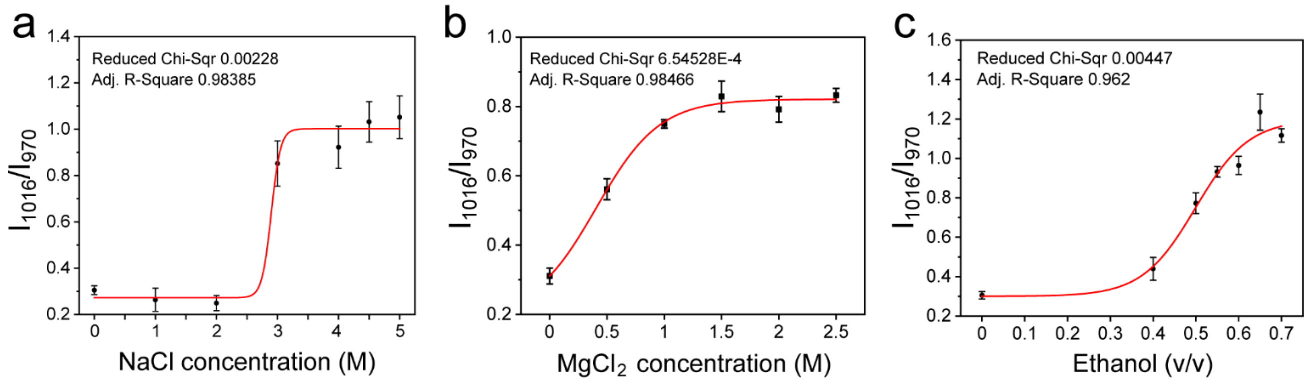

Figure S12. The ratio changes of 1016 cm^{-1} peak intensity to 970 cm^{-1} peak intensity with the concentration of inducers (data were supplied in the spectral fitting). a) The changes of I_{1016}/I_{970} of d(GC)$_8$ dsDNA induced by NaCl; b) The changes of I_{1016}/I_{970} of d(GC)$_8$ dsDNA induced by $MgCl_2$; c) The changes of I_{1016}/I_{970} of d(GC)$_8$ dsDNA induced by ethanol. The fitting function: $y=A_2+(A_1-A_2)/(1+\exp((x-x_0)/dx))$

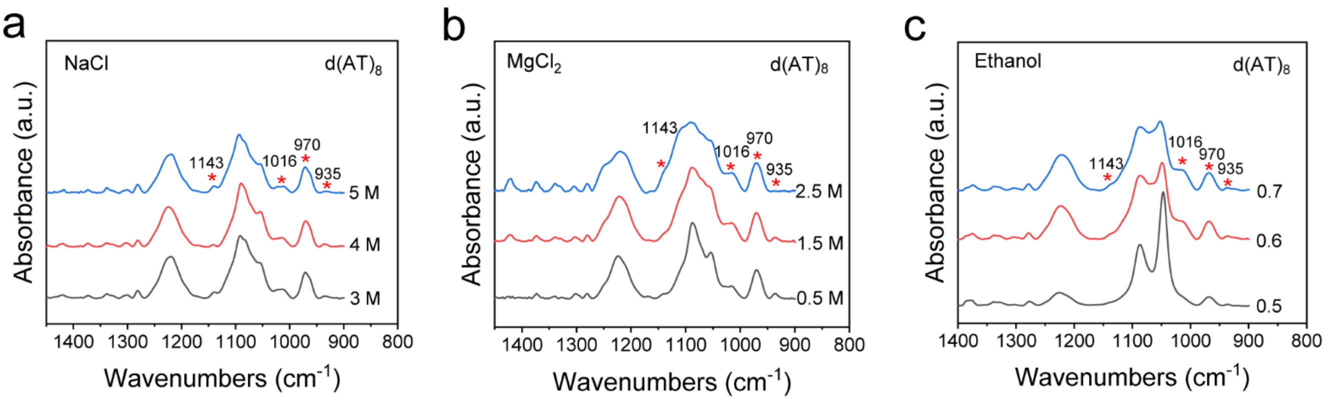

Figure S13. d(AT)$_8$ infrared spectra under different induction conditions. a) FTIR spectra of d(AT)$_8$ dsDNA treated with different concentrations of NaCl; b) FTIR spectra of d(AT)$_8$ dsDNA treated with different concentrations of $MgCl_2$; c) FTIR spectra of d(AT)$_8$ dsDNA treated with different volume ratios of ethanol.
